# Disabling sexualities: Exploring the impact of the intersection of HIV, disability and gender on the sexualities of women in Zambia

**DOI:** 10.4102/ajod.v2i1.50

**Published:** 2013-07-31

**Authors:** Anna Wickenden, Stephanie Nixon, Karen K. Yoshida

**Affiliations:** 1 Institute of Development Studies, University of Sussex, United Kingdom; 2 International Centre for Disability and Rehabilitation, University of Toronto, Canada; 3 Department of Physical Therapy and Graduate Department of Rehabilitation Science, University of Toronto, Canada; 4 Dalla Lana School of Public Health (Social and Behavioural Sciences and Health), University of Toronto, Canada

## Abstract

**Background:**

Women with a disability are often characterised as a homogenous social group consigned to a cultural stereotype with assumptions of dependence, asexuality and gender neutrality. Furthermore, there is a void of research about the experience of people with disabilities following diagnosis with HIV. Little is known about how HIV diagnosis intersects with disability and gender and how it shapes the experiences of intimacy and gender roles of those negotiating this intersection.

**Objective:**

The objective of this study was to explore how HIV, disability and gender shape the perspectives of HIV-positive women with disabilities regarding intimacy and gender roles.

**Methods:**

Twelve women in Lusaka, Zambia were recruited for in-depth semi-structured interviews to explore their experiences of having a disability and living with HIV. Interviews were conducted in English, Bemba, Nyanja and Zambian sign language. Descriptive and thematic analyses were conducted, followed by in-depth gender analyses of data relating to intimacy and gender roles.

**Results:**

Data analysis led to the identification of two main themes: the impact of HIV diagnosis on intimate relationships amongst the participants; and the disruption and renegotiation of gender roles. These findings demonstrate the loss of intimacy (often decided by the participants) and changes in women’s gender roles (infrequently decided by them).

**Conclusions:**

The narrow approaches to sexuality and HIV that reinforce misconceptions and stereotypes need to change. In their place should be inclusive and disability and sex-positive approaches that are informed by the diverse realities of women’s lives. Further research is needed to develop stronger evidence of the impact of HIV and disability on gender roles and sexuality.

## Introduction

The idea that women with disabilities have any sexuality may be an uncomfortable fact for much of society. This is reinforced by a narrow conceptualisation of sexuality as the sex act itself and the lack of research into the extraneous factors that impact and shape diverse sexualities. Several scholars have promoted thinking in terms of multiple sexualities and cautioned against oversimplifying and essentialising sexuality; yet the notion of a homogenous, unchanging sexuality continues to permeate much research and discourse (Helle-Velle 2004; Mama 2007; Oinas & Arnfred 2009; Tamale 2011).

The fact that HIV can impact on the sexualities of women with disabilities can create an uncomfortable proposition. HIV is intensely political and commonly associated with risk and sexual deviance. To think that HIV may have an impact on women with disabilities challenges many of the tenets that are fundamental to societies’ imagination and construction of ‘the disabled.’ In 1992, disabled American activist and author Anne Finger argued that:

‘Sexuality is often the source of our deepest oppression; it is also often the source of our deepest pain. It’s easier for us to talk about – and formulate strategies for changing – discrimination in employment, education and housing than to talk about our exclusion from sexuality and reproduction.’

Since the beginning of the HIV epidemic, Ikkaracan and Jolly (2006) and Edstrom (2010a, 2010b) argue, HIV and sexuality (particularly men having sex with men) have been intricately linked with the risk and vulnerability narratives that have accompanied the epidemic. Edstrom (2010b), Akwara, Madise and Hinde (2003) and Caravano (1991) contend that the way in which sexuality is understood and juxtaposed with HIV has reflected the intensely political nature of the discourse around HIV. The link between sexuality and HIV has focused on negative constructions of sex and sexuality or ‘bad sex’ (Jolly 2007; Rubin 1984). The negative association between HIV and sexuality has led to denial, blame and stigma surrounding HIV.

In contrast to the pairing of HIV and sexuality, disability and sexuality are issues that rarely come together in people’s minds (Meekosha 2004). In this article, we view disability through a social-model lens (Oliver 2004) informed by postcolonial critiques (Grech 2010). Common to all variants of the social model is the belief that ‘disability’ and ‘disablement’ are socio-political constructions. It is therefore the inhospitable physical and social environments combined with negative social attitudes that result in the systemic oppression, exclusion and discrimination of disabled people (Lang 2001).

The social model of disability facilitates the understanding of the process of the asexualising of disabled people, as described by Bonnie (2004) ‘Disabled adults have been infantilized, sterilised, prohibited from engaging in sexual activity and marriage, and excluded from social and leisure activities’. Meekosha (2010:4) and Thomson (1997) further explored the representation of the disabled as ‘asexual creatures, as freaks of nature, monstrous, the ‘Other’ to the social norm’.

Disabled women are often assumed to have no sexuality at all. Yet the image of disability may be intensified by gender, whereby women with disabilities may be viewed with intensified passivity and helplessness. The gendered experience of disability reveals patterns of difference of women which might be magnified following HIV diagnosis, as is often the case for non-disabled women (Edstrom 2010b).

HIV acts as a spotlight, exposing inequalities within and between countries and communities. The HIV epidemics are at their worst in regions where poverty and economic inequality is extensive and deep, and where gender inequality is pervasive. Tallis (2002) and Mahajan *et al*. (2008) argued that gender inequality is evident at all stages of the prevention-care continuum and can affect, amongst other things, the possibility of prevention, access to appropriate materials, information and resources, the quality of care received and the chances of survival.

Despite the emphasis on vulnerability in the HIV response, one of the world’s most ‘vulnerable’ populations – individuals with a disability – have until recently been almost entirely overlooked. Groce (2005) provided a succinct and powerful account of the intersection between HIV and disability, outlining the risk factors for disability and HIV through the life cycle. She emphasised that many of the established risk factors for HIV (poverty, illiteracy, stigma and marginalisation) are heightened amongst people with disabilities. A wave of important work has since emerged, which has investigated dimensions of HIV prevention for people with disabilities (Chireshe, Rutondoki & Ojwang 2010; Eide *et al*. 2011; Groce *et al*. 2013; Rohleder *et al*. 2012; Winningham *et al*. 2008). However, little is written about the HIV care, treatment and support needs of people with disabilities and who are HIV-positive, with most information currently residing in the form of reports developed by disabled people’s organisations as opposed to the peer-reviewed literature (Disability HIV and AIDS Trust [DHAT] 2011; STARZ Report 2009).

The purpose of this article is to explore the experiences of HIV-positive disabled women in relation to issues of intimacy and gender roles. This analysis derives from the Sepo Study, which is a project conducted jointly by researchers and activists at the University of Toronto and McMaster University in Canada, the University of KwaZulu-Natal in South Africa and the Disability HIV and AIDS Trust (DHAT), which is a Southern African disabled people’s organisation (and has an office in Zambia). The objective of the Sepo Study was to explore perceptions of stakeholders in Zambia regarding issues of health equity for people with disabilities and who are HIV-positive. This article presents an analysis of the accounts of female participants with such issues. It explores their experiences related to intimacy and gender roles.

## Methods

### Study Setting and Design

This qualitative, interpretive study was conducted in Lusaka, Zambia. Participants were women with disabilities and who are also HIV-positive. Within this group, diversity was sought in terms of impairments such as physical, hearing, visual and intellectual. Potential participants learned about the study through dissemination of recruitment information via posters and word-of-mouth within the HIV and the disability communities in Lusaka. Snowball sampling was also used, whereby participants were asked to share study information with other potential participants. Individuals interested in the study communicated with the study coordinator who was based in Lusaka. They then received additional information about the study purpose, procedures and safeguards for participants. Ethics approval was received from the University of Zambia, University of KwaZulu-Natal and the University of Toronto.

### Data Collection

In this cross-language, qualitative study (Temple 2002), in-depth, semi-structured interviews were conducted in one of four languages: English, Nyanja, Bemba and Zambian sign language. The research coordinator, fieldworkers and transcriptionists were all fluent in English, Nyanja and Bemba. Two of the fieldworkers were women with disabilities and two were HIV counsellors. In addition, two of the fieldworkers were certified Zambian sign language interpreters. For interviews conducted in sign language, the fieldworker conducted the interview in Zambian sign language whilst simultaneously vocalising the questions and responses to enable the digital recording and subsequent transcription. The fieldwork team was hired and trained specifically for this project. Training included an initial three-day orientation, a two-day mid-study training session and on-going support from the study coordinator.

The interview guide included questions to explore the experiences of living with both HIV and disability. The interviews explored the multiple experiences of the participants, as well as the meanings and interpretations that they attached to these experiences. Specific questions were also asked relating to sex, intimacy and partnership (see Box 1). Interviews were conducted at a private location chosen by the participant (usually in their homes). Data were stored on password-protected Universal Serial Bus (USB) keys until they could be transferred to an encrypted website. Data were collected from August 2010 to June 2011.

BOX 1 **Sample questions from the Sepo Study interview guide.**

**Table:** 

How is life different for you now that you have a disability and are also living with HIV? Do people have a different attitude toward you? How is life the same? What is your experience like within your family (now that you have both a disability and HIV)? Do they know you are HIV-positive? How did they react? Has this changed the way they treat you? Can you tell me any stories about how being HIV-positive has influenced your feeling of belonging or not belonging within your community? Have you ever had to fight for something that others have because you have a disability and HIV? How do you think your experience with HIV might be different from a non-disabled person who has HIV? How might it be the same? Do you feel that having a disability helps you handle HIV better in some ways than a non-disabled person? If so, how? How do you think your experience of disability might be different from other disabled people who are not HIV-positive? How might it be the same? Has HIV changed anything in terms of your relationships or intimacy? Some people say that having HIV is itself a disability. What do you think about that?

To facilitate collaborative coding and data analysis amongst the international research team, it was necessary to select one common language (English) in which all transcripts would be made available (Squires 2009). This was achieved through simultaneous transcription and translation; the transcriptionist listened to the digital audio file in the source language and transcribed it directly into English. Any vernacular content that did not translate easily into English was maintained in the source language in brackets in the transcript.

Various steps were implemented to increase cross-language trustworthiness and rigour (Squires 2009). Before the data was analysed, both the Zambian research coordinator and the fieldworker who conducted the interview reviewed all completed transcripts and compared them against the original audio file to ensure accuracy and completeness of translation (Lopez *et al*. 2008). The research team and fieldworkers participated in two data-analysis meetings where preliminary findings were presented and discussed in order to validate the results and provide additional contextual interpretations.

## Analysis

The analysis presented in this article was developed through a two-stage process. Firstly, the Sepo Study research team conducted a collaborative descriptive-analysis process of all data. For this stage, a coding framework was developed inductively and two members of the team coded each transcript. Data were organised using NVivo 9 (QSR International, USA). Each code was analysed descriptively and these preliminary results were vetted with the fieldwork team (Nixon *et al*. 2011). One set of findings related to sexuality and intimacy.

In the second stage, the co-authors of this article further analysed the data on sexuality and intimacy using a gender lens. Both original transcripts and coded data were revisited to examine how women in this study talked about:

- intimacy, partnership and sex
- themselves as sexual beings (or not)
- themselves as disabled beings (or not)
- family and community reactions to their HIV diagnosis.

How female participants’ roles and relationships were disrupted, changed or transformed following HIV diagnosis, and how participants were able to negotiate these consequences was explored. It was also noted as to how HIV, gender and/or disability (such as axes of inequalities) may have singularly or conjointly influenced these women’s experiences. Unique identifiers were used after each quote to indicate which participant had provided the data.

## Results

### Participants

Twelve women from Lusaka participated in this study (see Table 1). Six interviews were conducted in Nyanja, four in Bemba, one in Zambian sign language and one in English.

**TABLE 1:** Participants Characteristics.

Characteristic	Value
No. Of Participants:	12
Sex:	Female
Age Range:	29–61
**Type of impairment:**	
Hearing	1
Mobility	7 (1 also had intellectual impairment)
Visual	2
Intellectual	2 (1 also had physical impairment)

The participants described two main ways in which HIV, disability and gender had shaped their experiences of their sexualities. Firstly, data is presented to reflect the impact that HIV diagnosis and disclosure had on intimacy within their relationships. Secondly, participants’ views of the disruption and negotiation of gender roles that followed HIV diagnosis and disclosure are described.

### Impact on and changes to intimacy

During the interviews, participants were asked about the direct impact of HIV diagnosis on their intimate relationships. Participants were interviewed in the two sets (S) of in-depth interviews. It was through the descriptions of how participants contracted HIV that the limited agency of participants within their relationships was first indicated. Many of the women talked about their HIV status being caused by having partners who were promiscuous. One participant (P) (S1, P06) described how her boyfriend lied to her and was actually being ‘very promiscuous.’ Another participant described the nature of her relationship with her first husband:

‘Through my first husband, because he never used to, he was a truck driver, and was promiscuous … [*H*]e used to bring girlfriends in the house like that. Maybe he’d remove me from the bedroom, and he sleeps with the girlfriend in the sitting room.’ (S2, P08)

The accounts of these two participants, which are in contrast to descriptions of the relationships given by other participants, reinforce the importance of recognising that the impact of HIV diagnosis within intimate relationships is not experienced homogenously amongst women.

With the exception of one participant, all female participants who were interviewed were not in intimate relationships at the time of the interviews. Some had been left by their husband upon HIV diagnosis; others were widowed or never married. Only two of the female participants who had been in relationships at the time of diagnosis maintained those relationships after becoming HIV-positive.

The accounts of those participants whose relationships ended following HIV diagnosis were consistently described as being the decision of their partners. One participant described the reaction of her husband, who she told of her HIV diagnosis following the doctor’s advice that she ought to inform him:

‘So when I came home, after we ate, we even started laughing and chatting. That’s when I started telling him that, “Listen how it went at the clinic. They found me with the virus”. So my husband, I just saw, just there and then, he changed and got upset. “Found you with the virus, how? No, me, I don’t know what you are talking about”. So, um I started thinking ah! Ok. I then saw life in the house changing. My husband’s head that side, and my head that side [*Sleeping arrangement*]. We started differing in the home. So, I got back to the clinic. I told the doctor that, “No, this same husband, we’ve differed in the home. He has even decided to leave the house”’. (S1, P07)

Participants whose relationships had ended as a result of HIV diagnosis described being abandoned by their partners and in most cases, where there were children, participants were left to care for the children alone:

‘I started getting sick. So when I started getting sick, he [*husband*] told me that “You’re sickly. Why are you sickly?” I told him that, “Why am I sickly? It’s not my fault. I don’t know what I’m sick of. So it’ll be better for me to go to the clinic to get tested.” I got tested. I then told him that, “Listen, they’ve found me with such a disease.” [*HIV*] He said, “I can’t manage to live with a wife who is sickly. So it’s better I go and look for others who are not sickly. Remain with your ailments”. I told him that, “Ok, there’s no problem, only God knows”. That’s how he went.’ (S1, P10)

One other participant remained in a relationship. She described the impact of the HIV diagnosis on that relationship as being out of her control:

‘One, we are not intimate. We don’t sleep, we are not intimate. Two, he has refused to go for a test. He doesn’t take medication, says that, “It’s none of my business”. And, looking for food, he doesn’t put any effort. I start off alone; I go and search for food. Now imagine, I’m taking medication, I start off, going to look for food, but meanwhile, you’ve got somebody you are calling a husband in the house, and him, he has refused. How can you feel even if it were you, how can you feel?’ (S1, P07)

In this quote, the husband, when hearing of his wife’s HIV diagnosis, relinquished his responsibility for finding food for his family and left the woman with no choice but to take on this role as provider. The participant goes on to offer advice to disabled women, warning them not to be flattered by the attention of men and also cautioning them not to fear seeking treatment for HIV:

‘[*T*]he disabled, us the women should be careful. Don’t just see that this man has legs, he has liked me, I’m beautiful. Beware! Even me, I didn’t know, I thought that he has liked me, he’s showing me love. One, he saw that I had money. Two, he knew that this person will keep me forever. As I’m talking right now, the man does not do anything. But I go, despite my illness, I go and look for things. Fellow disabled, again if you are hiding [*your HIV status*], it will just bring you problems, and your relatives will have a loss. You’ll die, and life will still go on, while the medicine is even there, um. That’s what I just want to encourage the disabled.’ (S1, P07)

There is also evidence that some women were able to exercise agency in relation to their sexual relationships, sometimes consciously choosing abstinence. One participant, who had previously described the promiscuity of her husband, when asked about how she is able to take care of herself, explained:

‘Yes, I can take care of myself because I know that if I start being promiscuous, it will mean my CD4 count will go down, and I’ll be getting sick frequently … how can I take care of myself better, it is by avoiding men.’ (S2, P08)

The agency of this woman to make the decision of abstinence is also present in another interview, where a participant was asked if there is anything she did to improve her health status. She replied, ‘Support for HIV is not having boyfriends.’ (S1, P04)

Another participant (S1, P05) also explained that she decided there would no longer be an intimate sexual element to her marriage. Since being positively diagnosed with HIV, she and her husband had no longer been having an intimate relationship for fear that it would make the HIV worse. When asked if she was concerned her husband would seek sexual relationships outside of the marriage, she responded, ‘That’s his business. Now he should infect another person, that’s his business [*laughs*]. Now I don’t have any business with him.’ (S1, P05)

As such, some women actively decided to abstain from intimacy, whilst for others the decision was made for them.

### Disruption and renegotiation of gender roles and identity

The construction of femininity and the gender roles associated with it are intrinsically linked with the ability of women to fulfil expectations within the reproductive spheres of wife and mother. Female participants in this study repeatedly described the impact of the HIV diagnosis on such gender roles. Child rearing, care of household members and domestic duties were viewed as central to the construction of femininity and womanhood. Different participants described the impact of HIV diagnosis on each of these areas.

Participants who were of child bearing age and were not yet married spoke frequently about no longer being a desirable prospect for marriage following the HIV diagnosis:

‘There are so many men who are after me right now, but I could tell them straight without hiding that I’m HIV/AIDS. And you know what? They couldn’t believe, until I insist telling them that, “No, I’m a, I’m an HIV/AIDS.” And they could say, “Uh, no, you look nice.” And I could tell them, “Though I might look nice, still, I’m HIV/AIDS. And if you’re ready for me as an HIV/AIDS, then you can some-, you can, uh I can, you can, uh, agree to my proposal and I can accept it.” But maybe all of a sudden someone here starts, um, he disappears. Sometimes it really hurts me because maybe I, the one who proposes me, he’s a really, really good man and I could wish I could stay with that man. But, really do I have to, um, to tell him or to confide, to confide my history of HIV/AIDS? Cause the disability is being seen. When someone comes to propose me, they see that I’m on the wheelchair, but with HIV/AIDS it’s different … and sometimes if someone disappears, and I loved him very much, it pains me a lot.’ (S2, P08)

In this quote, it appears that the woman’s disability was not an issue for the men. However, the revelation of HIV and the associated stigma meant that the woman was no longer a marriage prospect. Other participants spoke of not pursuing marriage or remarriage as a choice, although the extent to which that choice was a ‘free’ choice is not always clear. One participant explained:

‘I don’t even want to re-marry … people, even when you find somebody that maybe you can spend your future with him, they refuse because of the same. They fear because you are [*HIV*] positive.’ (S2, P13)

For participants who were already married at the time of HIV diagnosis, only one talked about having a child after diagnosis. She described the reaction when she gave birth: ‘Me, the moment I just bore the child, they told me that, “Just this one. That’s all”’ (S1, P06). None of the other female participants in the study spoke of planning to have children or committing to marriage following HIV diagnosis.

The challenge that HIV diagnosis poses to the capacity of the female participants to care for household members and carry out domestic duties was articulated often throughout the study. How this impacts on family life and how this is negotiated varied widely. The majority of the participants described the challenge to fulfil their gender roles as a result of being diagnosed with HIV and having a disability, but one participant, with the support of her family, had been able to renegotiate her position and maintain her role within the household. When asked about how her HIV diagnosis had affected her role within the family, the participant noted, ‘As a wife, I have to execute my duties, and also as a mother, aah.’ (S2, P02)

HIV diagnosis and relationship breakdown in almost all cases further blurred the reproductive and productive roles of the participants. Female participants in the study often had to fulfil the role of provider for themselves, and in some cases for their children, as almost all the female participants were living in female-headed households. Their various impairments often presented as additional obstacles in fulfilling these roles. One female participant, whose husband left following her HIV diagnosis, explained that she had heard that he is also sick and unable to work and provide money for the children. She then reflected on the implications of this change in the role of provider within the family for her children:

‘I don’t manage as to what the children need. Sometimes they come and say, “Mummy, at school they want this thing.” If I don’t have, I tell them I don’t have. I say, “No, I don’t have. God will, time will come, he’ll give to us.” But I do complain because of the fact that I won’t manage to take care of the children alone, taking them to school on my own.’ (S1, P10)

Another participant provided a different perspective to the others who had almost exclusively described being HIV-positive and having disability as a double-burden in relation to accessing financial resources. She suggested that having HIV had a negative impact on an individual’s capacity to support themselves, but the fact that she had a disability meant that she was able to secure resources without engaging in sex work in comparison to other able-bodied women. This participant explained that she could protect herself more than the women who ‘go to the tavern’ who are not disabled because she is ‘able to go and beg.’ (S2, P11)

The burden of economic productivity also impacted on the perspectives on the role of government. One participant explained that she believes the state should sometimes intervene not only after HIV diagnosis, but in order to prevent contracting HIV in the first instance, especially for people with disabilities:

‘The government needs to support us in every way, especially for us who are HIV/AIDS. And for those maybe who are not HIV/AIDS. Yes maybe because of poverty that has made us, many of our disabled people, uh to engage themselves with men so that they give them money and at the end of the day you find yourself with HIV/AIDS. If they could do something uh, to keep busy, not to be thinking of men, who could do nothing but bring us some virus like this.’ (S2, P10)

Gender roles are also formed and shaped through interactions between individuals and their social world. They are also pivotal to the construction of identity and experience in relation to gender and sexuality. Participants repeatedly described how their HIV diagnosis impacted on their identity and on social relations due to the reactions and attitudes of community and family members. Negative comments from families were characterised as particularly ‘hurtful’ and difficult to receive. Some participants would only disclose HIV status to family members that they thought should know or would be supportive. Reactions from the community were also often characterised as painful and negative. One female participant described the way the community reacted to her diagnosis:

‘No, we don’t feel good because what they normally say is offending. They say that, “This person also, she is disabled and she adds on this disease. Can’t she just stay? Also the doctor that gives her medication, if only he could give her one for killing her. I heard this for myself.’ (S2, P11)

The majority of female participants experienced ostracism from some family members or within their communities. However, there were also examples of support from community members and family members:

‘Even when I say that I’m ill, they [*family*] are fast to take care of me, yes. When I say, “Ah, I’m not feeling well. What is the matter?” Fast fast, they take care of me, concerning issues of hospital.’ (S2, P08)

The participants who described positive reactions from their partners, families or communities were more likely to also provide examples of how they negotiated their sexualities and gender roles in a broader context. Overall, the analysis demonstrates that HIV diagnosis, gender and disability may singularly or jointly influence the experiences of women in relation to intimacy and gender roles within the household and family. However, how this impact manifests and how the participants negotiate it varied significantly and may be linked to factors that existed prior to HIV diagnosis.

## Discussion

This is the first article that explores how the intersection of disability, HIV and gender amongst women with disabilities and who are living with HIV has shaped their perspectives on their sexualities. More broadly, this article is amongst the first to present empirical data from people with disabilities who are positively diagnosed with HIV. As such, it extends the existing literature on HIV prevention amongst disabled communities by refocusing on the experience of living with HIV, which has been largely overlooked to date (Groce *et al*. 2013). In particular, our analysis led to the identification of two main themes: the impact of HIV diagnosis on the intimate relationships of the participants and the disruption and renegotiation of gender roles. These findings challenge misconceptions about asexuality and genderless identity amongst women with disabilities.

### Challenging stereotypes of asexuality

Participants’ accounts challenge the assumptions of disabled people being asexual and without gender. Numerous authors, including Milligan and Neufeldt (2001) and Bonnie (2004), have described how society tends to dismiss sexuality as a fundamentally important factor in the lives of disabled people:

’[*M*]any people assume we are asexual, often in order to hide embarrassment about the seemingly incongruous ideas that such “abnormal” people can have “normal” feelings and relationships.’ (Morris 1989:80)

Bonnie (2004) argued that there has been a taboo in many societies around discussing the sexuality of disabled people, since sex has been associated with marriage and procreation and disabled people are often not expected to experience either. Assumptions around the asexuality of persons with disabilities can manifest in different ways, including assumed inability to engage in sexual activities and lack of attraction to others. This can be compounded by the expectation that people with disabilities are dependent on others and not seen as productive individuals who could contribute to partnerships. Participants’ accounts in this analysis challenge these assumptions, as the women portray full lives that include intimate relations and productive roles as wives and mothers.

### Implications for Intimacy

The women in this study describe how HIV diagnosis had a significant impact on the expression of their sexualities through intimacy and intimate relationships. How participants negotiated the impact of HIV and disability with multiple situational factors reflects the diversity of the lives of female participants prior to HIV diagnosis. The limited agency to renegotiate their roles within intimate relationships is described often, with only two female participants maintaining relationships after being positively diagnosed with HIV and only one of those continuing physical intimacy within that relationship. The impact of restrictions on intimacy and the breakdown of relationships can be far reaching. Participants whose relationships ended following HIV-diagnosis characterised this decision as being the choice of their partners. Although several participants also describe ways in which they are re-negotiating their roles within their relationship or household, this is not the norm amongst participants in this study. They often described being abandoned and left to provide for themselves and their children, an experience also seen amongst able-bodied women upon being positively diagnosed with HIV. This finding allies disabled and non-disabled women in the struggle for gender equity, as it relates to HIV stigma. There also appears to be limited awareness about strategies for decreasing the risk of HIV transmission during physical intimacy and in pregnancy, reinforcing the call for HIV education amongst people with disabilities (DHAT 2011; Groce 2005; Rohleder *et al*. 2012).

### Human immunodeficiency virus, gender and disability

The disabled women in this study narrate their experiences in gendered terms. The impact of gender, disability and HIV on the female participants supports the reviewed literature about gender, sexuality and disability. The literature argues against a narrow, homogenous or static understanding of gender, sexuality or disability and instead emphasises the impact of culture and society in shaping how gender, sexuality and disability are experienced and the central role they play in maintaining power relations in society (Helle-Valle 2004; Mama 2007; Morris 1989; Oinas & Arnfred 2009; Shakespeare 2000; Tamale 2011). It also extends this research base by exploring how HIV can impact on these dynamics. The female participants’ abilities to negotiate roles as mother or wife are impacted by both HIV and by stereotypes related to disability. Gender inequalities appear to be further entrenched through the sexual dimension of living with HIV and the social dimensions of reproductive and familial care responsibilities. Despite these challenges, women demonstrate resilience in creating supportive environments for themselves and their children. These findings challenge assumptions about the gender neutrality of women with disabilities, as evidenced by the narratives of how these disabled women have experienced gender constructions throughout their lives.

## Limitations

The in-depth interviews on which this analysis is based were not singularly focused on gender or sexuality. As such, the training provided to fieldworkers did not focus as heavily on how to collect sensitive data on sexuality, as would have been the case in a study primarily looking at this topic. As such, it is possible that limitations of the data relating to sexuality may reflect not only on the possible hesitation of the participants to reveal insights, but also on the potential hesitation of the fieldworkers to probe into this topic during interviews (Gune & Manuel 2011).

An additional limitation relates to the theories of power and knowledge production. When writing about research of ‘African’ sexualities, Tamale (2011) reminds us that the language of Western colonialists has dominated sexual discourses; as a result, rich cultural connotations and ambiguities can be lost in translation. To mitigate this impact, the authors attempted to remain close to the data and to relate it to the theoretical perspectives advanced by both developed and developing world scholars across the fields of HIV, disability and gender.

## Conclusion

Overall, this study illustrates how assumptions regarding the sexualities of disabled women permeate the lives of the participants and shape their interactions within their relationships, their community and family. As such, approaches to sexuality and HIV that consider only the impact of health need to be expanded in order to consider the many ways in which sexuality is part of the lives of individuals, both within heterosexual relationships between non-disabled women and men, and those who are not. The findings call for increased recognition of the importance of sexuality in all individuals’ lives, noting that sexuality relates not only to health and reproduction, but is also interconnected with the broader notions of wellness, integration and inclusion. Policy, programme and research approaches to disability and HIV can be strengthened by becoming inclusive, gendered and by avoiding essentialisms. Responses can be informed by the complex and diverse realities of women’s lives, emphasising diverse abilities, sexualities and capacities.

Further research is needed to explore the different ways in which both masculinity and femininity are constructed and are impacted by HIV and disability, whilst recognising that gender and sexualities permeate all elements of identity. Little is known about the impact of HIV diagnosis on the sexualities of men with disabilities. However, literature related to men, masculinities and disability discusses the dislocation of masculinity experienced by men with disabilities. Meekosha (2004), Nario-Redmond (2010) and Shakespeare (2000) argued that men with disabilities are often feminised in the way that they are constructed in the public sphere. The impact of HIV, disability and gender on the masculinity of the participants may be more apparent in the obstacles that they experience in relation to employment, social status and participation in society and the impact on independence, self-sufficiency, ability to provide for their families and to their capacity to be self-determining. These are important areas of enquiry going forward. Finally, there is a need for research that explores the positive sides of sexuality, particularly amongst those for whom gender norms and stereotypes obstruct opportunities to seek pleasure and fulfilment. By building this evidence base, we can further challenge the damaging stereotypes about gender and ability that typically inform approaches to HIV.
